# Comparison arthroscopic reconstruction and percutaneous reconstruction of ankle lateral ligament for chronic ankle lateral instability: A protocol for a meta-analysis of comparative studies

**DOI:** 10.1097/MD.0000000000031926

**Published:** 2022-11-11

**Authors:** Huiming Hou, Ming Zhou, Xing Zhou, Wenxuan Guo, Rujie Zhuang, Hong Yin, Jinlei Li

**Affiliations:** a Nanchang Hongdu Hospital of Traditional Chinese Medicine, Nanchang City, China; b Department of Orthopaedics, The First Affiliated Hospital of Zhejiang Chinese Medical University, Hangzhou City, China; c The First Clinical College, Zhejiang Chinese Medical University, Hangzhou City, China; d Kunming University of Science and Technology Hospital, Kunming City, China; e Kunming Municipal Hospital of Traditional Chinese Medicine, Kunming City, China.

**Keywords:** arthroscopic reconstruction of ankle lateral ligament, chronic ankle lateral instability, percutaneous reconstruction of ankle lateral ligament, protocol

## Abstract

Ankle sprains occur very frequently in daily life, but people who do not pay attention to them and do not receive proper diagnosis and treatment are very prone to develop chronic ankle lateral instability (CALI) at a later stage. For CALI where conservative treatment has failed, reconstruction of the lateral collateral ligament of the ankle can achieve satisfactory results, but there are various and controversial ways of ligament reconstruction. While percutaneous reconstruction of ankle lateral ligament (PLCLR) needs to be performed repeatedly under fluoroscopy, total arthroscopic reconstruction of ankle lateral ligament (ALCLR) is increasingly recognized by experts and scholars for its minimally invasive and precise characteristics, and has achieved good clinical results. Therefore, it is imperative that a meta-analysis be performed to provide evidence as to whether there is a difference between ALCLR and PLCLR in the treatment of CALI.

**Methods:**

We will search articles in 7 electronic databases including Chinese National Knowledge Infrastructure, Wanfang Data, Chinese Scientific Journals Database, Chinese databases SinoMed, PubMed, Embase, and Cochrane Library databases. All the publications, with no time restrictions, will be searched without any restriction of language and status, the time from the establishment of the database to September 2022.We will apply the risk-of-bias tool of the Cochrane Collaboration for Randomized Controlled Trials to assess the methodological quality. Risk-of-Bias Assessment Tool for Non-randomized Studies was used to evaluate the quality of comparative studies. Statistical analysis will be conducted using RevMan 5.4 software.

**Results:**

This systematic review will evaluate the functional outcomes and radiographic results of ALCLR in the treatment of CALI.

**Conclusion:**

The conclusion of this study will provide evidence for judging whether ALCLR is superior to PLCLR for treatment of CALI.

**Trial registration number:**

CRD42022362045.

## 1. Introduction

Up to 50% to 70% of patients may develop chronic ankle lateral instability later after ankle sprains.^[1]^ The most frequent injury from sprains is to the lateral collateral ligament of the ankle, including the anterior talofibular ligament (ATFL), which has the highest rate of injury, and the calcaneofibular ligament (CFL), which has a 50% to 75% probability of injury.^[2]^ Acute ankle sprains that are not properly diagnosed and treated late will pose a risk of ankle instability, which can lead to chronic ankle instability, resulting in pain and limited motion and accelerated joint degeneration.^[3–5]^ With increasing attention to ankle sprains, early treatment with more standardized treatment can effectively reduce the probability of late ankle instability.^[6,7]^ However, due to the degree of injury to the lateral collateral ligament of the ankle, many patients still develop chronic ankle instability, and then surgery is required to restore ankle stability.^[8,9]^

Since most of the ligaments in patients with chronic lateral ankle instability cannot be repaired, surgical treatment usually requires reconstruction of the ATFL and CFL ligaments to restore joint stability.^[10–12]^ Currently, due to the prevalence of minimally invasive concepts, the main methods of ligament reconstruction are percutaneous reconstruction and arthroscopic reconstruction.^[13–15]^ Percutaneous reconstruction often requires repeated x-ray fluoroscopic confirmation, which will undoubtedly increase the operative time and aggravate the damage to the medical staff and patients.^[16,17]^ However, due to the rapid development of arthroscopic techniques, more and more experts and scholars have mastered the all-scopic reconstruction of ATFL and CFL ligaments.^[18,19]^ Arthroscopy allows for the combined management of intra-articular injuries, such as cartilage damage and free body formation due to repeated impingement; in addition, arthroscopy allows for the precise localization of the ligament footprint area, reducing the number of x-ray fluoroscopies and avoiding increased operative time and physical damage.^[20,21]^

However, due to the increasing number of relevant studies published in recent years, the lack of high-level guidelines and evidence-based medical evidence has seriously affected clinical decision making and application. In this study, we attempted to conduct a meta-analysis of relevant studies to evaluate and compare the functional outcomes and complication rates of arthroscopic reconstruction of ankle lateral ligament with percutaneous reconstruction of ankle lateral ligament for chronic ankle lateral instability, and our findings are expected to provide a reference for guiding future treatment options.

## 2. Methods

### 2.1. Study registration

We have prospectively registered this research at the international prospective register of systematic reviews-Registration number: CRD42022362045. We performed this protocol based on the Preferred Reporting Items for Systematic Review and Meta-analysis Protocols (PRISMA-P) statement guidelines.^[22]^

### 2.2. Inclusion criteria

#### 2.2.1. Type of participants.

The participants diagnosed as chronic ankle lateral instability will be included regardless their country, ethnicity, sex, occupation and mechanism of injury.

#### 2.2.2. Type of interventions.

In the experimental group, all patients received arthroscopic reconstruction of ankle lateral ligament. In the control group, all patients received percutaneous reconstruction of ankle lateral ligament.

#### 2.2.3. Type of outcome measurements.

##### 2.2.3.1. Primary outcomes.

American Orthopaedic Foot and Ankle Society midfoot score will be defined as the primary outcomes to assess the function and stability.

##### 2.2.3.2. Secondary outcomes.

Visual analog scale, Tegner score, complications will be defined as secondary outcomes.

#### 2.2.4. Type of studies.

We will include comparative studies which published in Chinese or English, such as randomized controlled trials, retrospective studies and cohort studies. Review, case reports, experimental studies, expert experience, animal studies and conference abstracts will be excluded.

### 2.3. Search strategy

CNKI, Wanfang, VIP, CBM, PubMed, Embase, and Cochrane Library databases were searched for this study. using the keywords “ankle,” “ankle instability,” “ankle ligament injury,” “arthroscopy,” “percutaneous,” “reconstruction,” and “surgery.” The search strategy in PubMed is shown in Table 1. In addition, the reference lists of previously published systematic reviews of Ligament Reconstruction for ankle instability were manually examined for further pertinent studies.

**Table 1:** Pubmed database search strategy.

Search number	Items
1	“ ankle instability ”[Mesh]
2	ankle [Title/Abstract]
3	ankle instability[Title/Abstract]
4	ankle ligament injury[Title/Abstract]
5	1 OR 2 OR 3 OR 4
6	arthroscopic[Title/Abstract]
7	percutaneous[Title/Abstract]
8	reconstruction[Title/Abstract]
9	surgery[Title/Abstract]
10	6 OR 7 OR 8 OR 9
11	5 AND 10

### 2.4. Study selection

**Figure 1.**
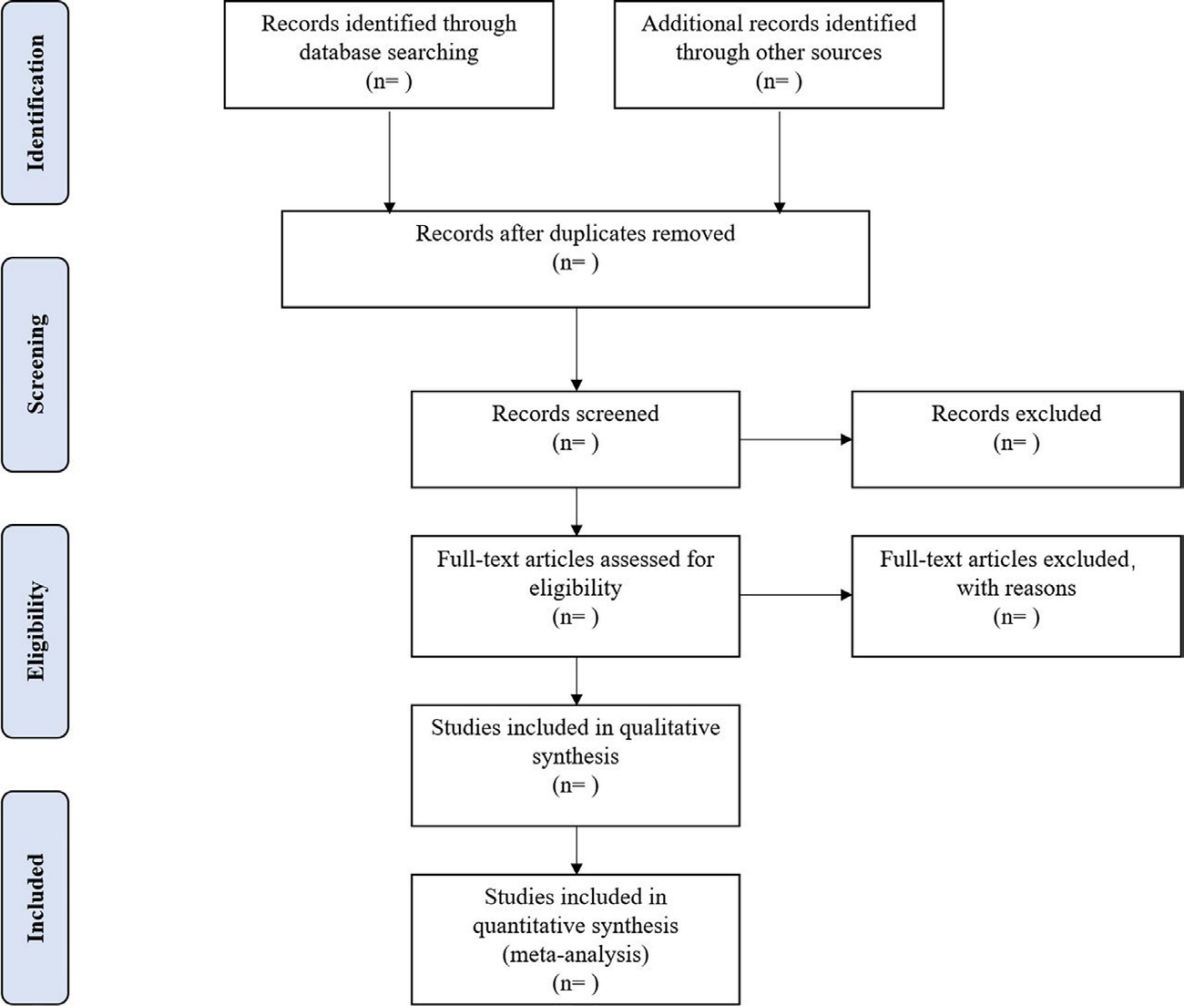
Flowchart of literature selection.

Different researchers (XZ and HH) will separately screen the titles and abstracts of records acquired potential eligibility which comes from the electronic databases. The obtained literature is managed by Notoexpress, irrelevant and duplicate articles are excluded by reading the title and abstract, full texts screening and data extraction will be conducted afterward independently, and finally selected according to the inclusion criteria, any disagreement will be resolved by discussion with the third author (JL) until consensus is reached or by consulting a third author. PRISMA-P flowchart Will be used to show the selection procedure (Fig. 1).

### 2.5. Data extraction and management

The following data were extracted: lead author; publication year; country of origin; study design; sample size; age; injury type; reconstruction technique; outcome measures and complications. Any differences of opinion will be resolved through group discussion or consultation with a third reviewer. When relevant data is not reported, we will contact the author via email or other means to obtain missing data. The PRISMA flow diagram will be filled out after the screening study is completed to provide specific information.

### 2.6. Risk of bias assessment

Two independent (XZ and WG) investigators evaluated the quality of the included studies. The Cochrane Collaboration Risk of Bias Tool was used to evaluate the quality of the randomized controlled trials. The methodological quality of the non-randomized studies was assessed using the Risk-of-Bias Assessment Tool for Non-randomized Studies. The level of evidence was assessed according to the Oxford Centre for Evidence-based Medicine Levels of Evidence.

### 2.7. Data synthesis

Statistical analysis will be conducted using RevMan 5.4 software (Cochrane Collaboration). The mean difference will be used as the effect analysis statistic for continuous variables, while the risk ratio will be used as the effect analysis statistic for categorical variables. We will also calculate 95% confidence interval for each statistic, and summarize statistical heterogeneity among summary data using the *I*^2^ statistic. Cases with *I*^2^ ≤ 50% will not be considered to have significant heterogeneity, thus a fixed-effects model will be applied for meta-analysis. In cases where there is statistical heterogeneity among studies, we will further analyze the source of heterogeneity. A random-effects model will be used to pool the data, after excluding the obvious source of clinical heterogeneity, and in cases where obvious clinical heterogeneity exists, the researchers will perform subgroup, sensitivity or only descriptive analyses. Study-specific and pooled estimates will be graphically presented using forest plots, and *P* < .05 considered statistically significant.

### 2.8. Subgroup analysis

Subgroup analysis according to the age, injury type and the classifications for chronic ankle lateral instability will be performed to find the source of heterogeneity when significant clinical heterogeneity is observed.

### 2.9. Sensitivity analysis

Sources of heterogeneity were assessed by sensitivity analysis, by excluding studies of low quality or small sample size, if the heterogeneity did not change significantly, the results were robust. otherwise, the excluded studies may have been source of heterogeneity.

### 2.10. Publication bias

In this study, fewer than 10 included studies were evaluated for publication bias using funnel plot, otherwise Egger regression test would be used.^[23,24]^

### 2.11. Ethics and dissemination

No ethical approval is required because the study will be a review of literature and will not obtain data from a single patient. We will publish our findings through a peer-reviewed journal.

## 3. Discussion

The purpose of this study was to comparatively evaluate the functional outcomes and complications of arthroscopic reconstruction of ankle lateral ligament and percutaneous reconstruction of ankle lateral ligament in the treatment of CAIL. With the maturation of arthroscopic techniques, more and more experts and scholars are using arthroscopic minimally invasive and precise treatment of ankle instability. To the best of our knowledge, this study integrates the most recent and comprehensive clinical evidence in this field, hoping to provide patients and clinicians with useful and high-grade evidence-based medicine.

## Author contributions

**Conceptualization:** Xing Zhou, Jinlei Li.

**Data curation:** Huiming Hou.

**Formal analysis:** Ming Zhou, Hong Yin.

**Funding acquisition:** Rujie Zhuang, Jinlei Li.

**Investigation:** Huiming Hou, Xing Zhou, Hong Yin.

**Methodology:** Huiming Hou, Wenxuan Guo.

**Resources:** Ming Zhou, Xing Zhou, Wenxuan Guo.

**Software:** Ming Zhou, Xing Zhou.

**Supervision:** Ming Zhou, Jinlei Li.

**Writing – original draft:** Huiming Hou, Xing Zhou.

**Writing – review & editing:** Rujie Zhuang, Jinlei Li.
